# Retrotransposons in pluripotent stem cells

**DOI:** 10.1186/s13619-020-00046-4

**Published:** 2020-06-02

**Authors:** Jingwen Wang, Junjiu Huang, Guang Shi

**Affiliations:** 1 School of Life Sciences, SunYat-sen University, Guangzhou, 510275 P. R. China; 2 Key Laboratory of Reproductive Medicine of Guangdong Province, School of Life Sciences and the First Affiliated Hospital, Sun Yat-sen University, Guangzhou, 510275 China; 3 Key Laboratory of Reproductive Medicine of Guangdong Province, Third Affiliated Hospital of Guangzhou Medical University, Guangzhou, 510150 China

**Keywords:** Retrotransposon, Pluripotent stem cells, Pluripotency, Epigenetic regulation

## Abstract

Transposable elements constitute about half of the mammalian genome, and can be divided into two classes: the class I (retrotransposons) and the class II (DNA transposons). A few hundred types of retrotransposons, which are dynamic and stage specific, have been annotated. The copy numbers and genomic locations are significantly varied in species. Retrotransposons are active in germ cells, early embryos and pluripotent stem cells (PSCs) correlated with low levels of DNA methylation in epigenetic regulation. Some key pluripotency transcriptional factors (such as OCT4, SOX2, and NANOG) bind retrotransposons and regulate their activities in PSCs, suggesting a vital role of retrotransposons in pluripotency maintenance and self-renewal. In response to retrotransposons transposition, cells employ a number of silencing mechanisms, such as DNA methylation and histone modification. This review summarizes expression patterns, functions, and regulation of retrotransposons in PSCs and early embryonic development.

## Background

Pluripotent stem cells (PSCs) are cells that have potential to differentiate into various cells and tissues. The most important properties of PSCs are self-renewal and differentiation into adult stem cells or multiple somatic cells during development (He et al., 2009; Hackett & Surani, 2014). Most researches were focused on cell differentiation, induced PSC (iPSC) reprogramming efficiency, and disease modeling with emphasis on clinical therapies, thus conditions of PSCs are important (Ding et al., 2013; Avior et al., 2016; Mora et al., 2017; Bernareggi et al., 2019). In PSC culture, heterogeneity is a key issue that remains to be unresolved (Hayashi et al., 2019). Apart from researches about gene expression and epigenetic modifications that are specific to PSCs (Bar-Nur et al., 2011; Kim et al., 2012; Stelzer et al., 2013), more and more scientists have begun to focus on transposon elements, which make up almost half of the mammalian genome and were considered “junk” DNA (Chen et al., 2008). Retrotransposons are the most mobile class of transposon elements and studies have demonstrated that they are transcribed more in early embryos and PSCs than somatic cells (Kigami et al., 2003; Zhou & Smith, 2019).

Early mammalian embryos undergo widespread epigenetic reprogramming accompanied by chromatin re-establishment and organization (chromatin interaction) (Xu & Xie, 2018). Studies have shown that cis-regulatory elements (such as promoters, enhancers, and super-enhancers) and trans-factors (such as transcriptional factors and epigenetic effectors) can drive embryonic development. It is worth mentioning that some pluripotency transcriptional factors (such as OCT4, SOX2, and NANOG) control stem cells fate by regulating pluripotency maintenance and lineage differentiation repression, and contributing to the open chromatin architecture of stem cells (Ahmed et al., 2010; Mulas et al., 2018; Bakhmet et al., 2019). Recently, more and more studies have identified that retrotransposons also play important roles in PSCs and embryos, and some factors regulating the elements have been identified (Coluccio et al., 2018; Percharde et al., 2018). Combined with the fact that pluripotency transcriptional factors bind retrotransposons and regulate their activities in PSCs, it is logical to assume that retrotransposons have functions in embryogenesis, maintaining PSCs pluripotency and so on. In this review, we report on recent advances in the study of retrotransposons in mice and humans with a focus on the expression patterns, specific roles in distinct development stages, and important regulation mechanisms.

## Retrotransposons in genomes

**Fig. 1:**
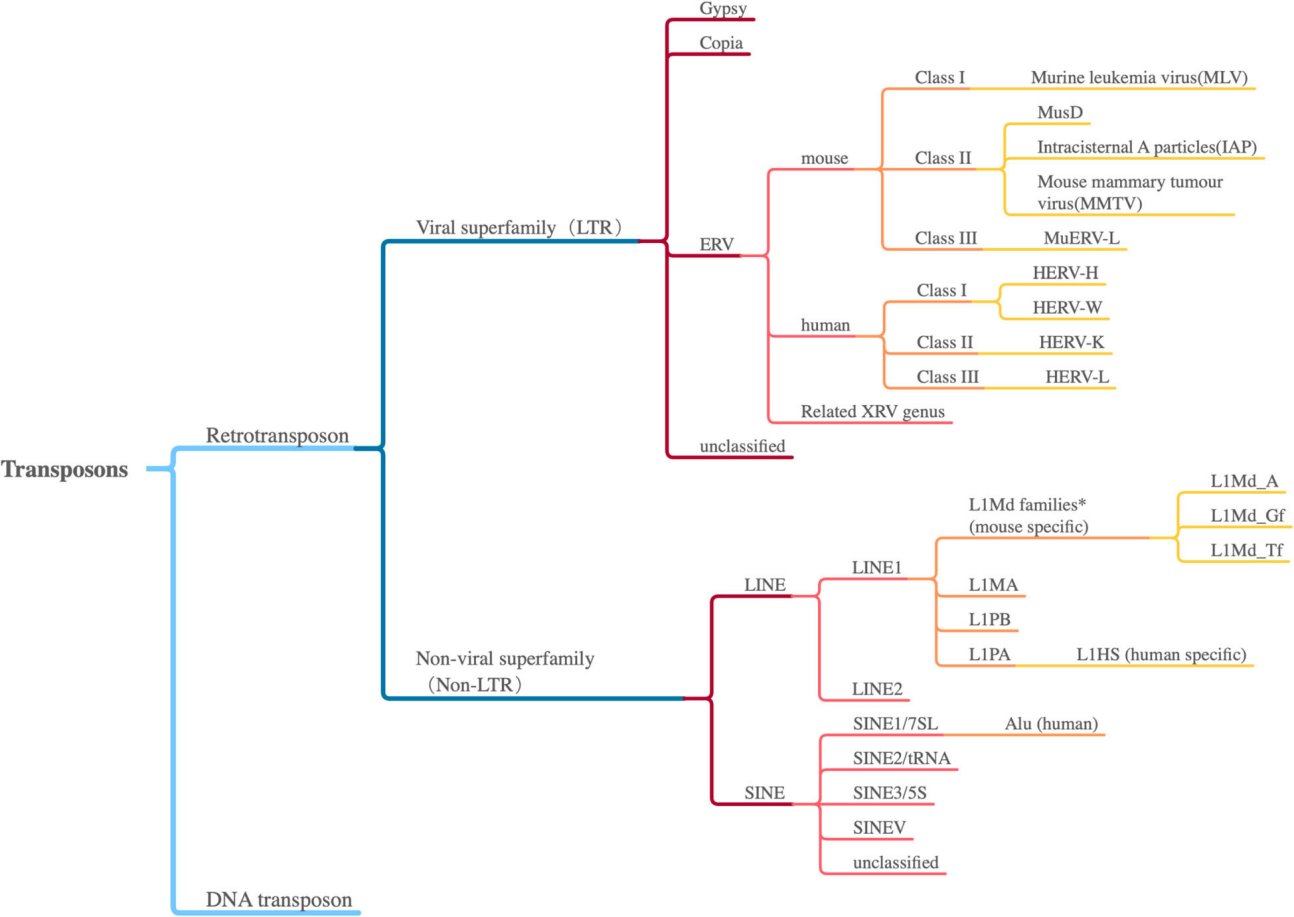
Retrotransposon classification. A tree diagram of transposons. Well-known classes and subclasses are listed, and mouse/human specific families are highlighted. Transposons are divided into two classes by the type of transposition. Retrotransposons are classified into two types according to the encoding transposase. ERVs are the most popular elements studied in LTRs. In LINE1 subfamilies, L1MA4–1 and L1PB3–1 are already extinct, and L1PA evolved as a single lineage

Transposable elements (TE), also known as transposons, were first discovered in 1948 by geneticist Barbara McClintock (Mc, 1948; Mc, 1950). She found certain chromosomes can change their position from one generation to the next and can even be turned on or off in different cell culture stages and environment conditions. In the 1960s to 1970s, this kind of “jumping” DNA sequences were also found in bacteria. As reported in whole genome sequencing projects, almost half of mammalian genomes and approximately 90% of the maize genome was made up of TEs (Lander et al., 2001; Schnable et al., 2009). There are two classes of TEs: the class I (retrotransposons) and the class II (DNA transposons). Class I TEs are transcribed to RNA, then reverse transcribed to cDNA, and integrated in the genome by a “copy and paste” mechanism. Class II TEs can encode transposase enzymes to “cut and paste” directly. The detailed classification is shown in Fig. 1.

Retrotransposons are commonly classified into two subclasses with variant characters and functions. Subclass I consists of viral retrotransposons characterized by long terminal repeats (LTRs) on both terminals. They encode reverse transcriptase and change position like retroviruses, but lack a functional envelope gene (Env). They constitute about 8% of the human genome and approximately 10% of the mouse genome (Mameli et al., 2007). The most abundant and more active subclass in human called endogenous retroviruses (ERVs), a type of LTR retrotransposon, may originate from retroviruses and constitute 5–8% of human genome (Nelson et al., 2004). Another endogenous retroviral-like LTR element was identified in mice, such as intracisternal A particle (IAP), which repeated 1–2000 times in mouse genome, and has been found to transpose in mouse tumor cells and germ cells of a few mouse strains (Kuff & Lueders, 1988).

Subclass II consists of non-viral retrotransposons, also known as non-LTR TEs (Feng et al., 1996). They can be further divided into two subgroups by the length of elements. The first subgroup is long interspersed nuclear elements (LINE), which has an average length of 7000 base pairs and is widespread in many eukaryotes, comprising 21% of the human genome. The structure of LINEs contains a 5’UTR, two non-overlapping open reading frames (ORF), a 3’UTR, and a 3′ poly (A) tail. The first ORF encodes a nucleic acid binding protein (Holmes et al., 1992) and the second ORF of LINEs may encode reverse transcriptase (Sakaki et al., 1986), and there is an endonuclease domain at the ORF2 N-terminus, which helps to cleave the target site for insertion (Moran et al., 1996). Most LINE elements are 5′ truncated, suggesting that 5′ sequence is not indispensable while 3′ poly (A) tail is critical in transposition (Feng et al., 1996). LINE-1(L1), a subgroup of LINEs, can be found in all mammals and is the only activated autonomous element in human genome with an estimated 500,000 copies (Belancio et al., 2008). The second subgroup of non-LTR retrotransposons short interspersed nuclear elements (SINE) is 50–500 base pairs in length. They are non-coding transposons and utilize other TE reverse transposases for reposition. There are over 500,000 copies of Alu SINE elements in human genome and suspected to cause various diseases by disrupting gene sequences.

## Retrotransposons in early embryo development

**Fig. 2:**
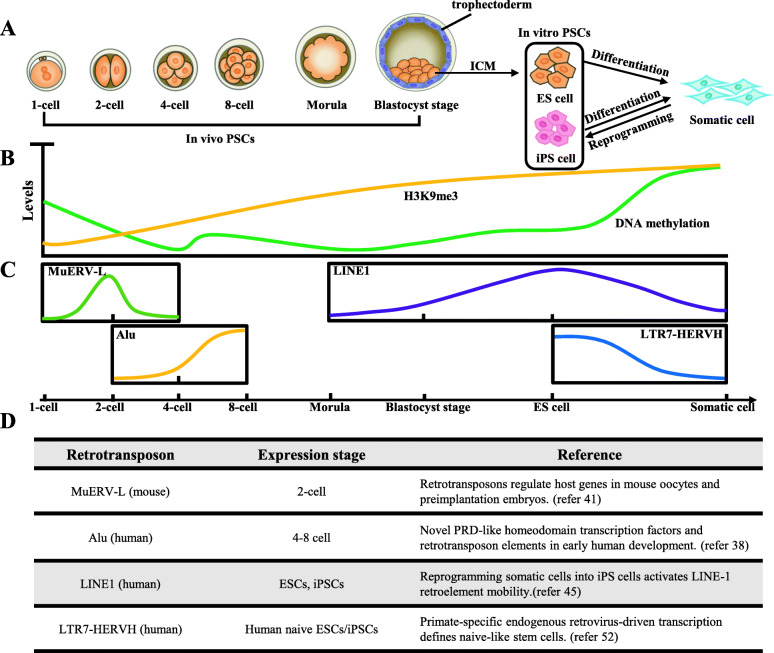
Epigenetic modification and retrotransposon expression changes in embryogenesis. **a** Different stages of embryonic development process are showed (from left to right): 1-, 2-, 4-, 8-cells, morula, and blastocyst stages. Embryonic stem cells (ESCs) are derived from the inner cell mass (ICM) of the early blastocysts, and have similar developmental potential as ICM. Inducible pluripotent stem cells (iPSCs) come from differentiated somatic cells and together with ESCs comprise in vitro PSCs; **b** DNA methylation (green) and H3K9me3 (yellow) levels decrease after fertilization, then increase to normal levels in somatic cells; **c** The expression of retrotransposon subclasses changes in the embryonic development process, and some typical elements are shown such as MuERV-L(green), Alu(yellow), LINE1(purple), and LTR7-HERVH(blue); **d** The summary of expression patterns of retrotransposons demonstrated in (**c**) and related references

There is a remarkable change in DNA methylation from sperm to the zygote. DNA methylation level increases progressively during embryo development and finally reaches normal level in post-implantation embryos (Fig. 2a and b). This process is accompanied by changes in retrotransposon expression.

For non-LTR elements, studies have shown that L1 and IAP retrotransposons become reactivated from both parental genomes after fertilization (Fadloun et al., 2013). In mice, over 85% LINEs have significant translational changes and about 18% LINEs display a change in methylation levels (more than 45%), which are concentrated in *Mus musculus* domesticus L1 retrotransposons (L1Md_Tf and L1Md_Gf classes), suggesting specific roles during fertilization (Smith et al., 2012). L1Md families can express at the onset of germ cell meiotic prophase I (MPI) and are the most abundant and active TEs in the mouse genome (Gaysinskaya et al., 2018). Further research also indicates that each of the three L1Md classes (A, Gf, and Tf) has much higher expression in 2-cell embryonic stage than in mouse ESCs (mESCs). Notably, L1Md_A can even be detected at 8-cells stage and progressively decreases till 16-cells stage (Jachowicz et al., 2017). Most of the newly generated L1 DNA copies are unstably integrated into the genome, while some of them can stably insert to the genome leading to genetic diseases, such as aberrant splicing of glycine receptor beta subunit mRNA and exon skipping caused by the L1 element retrotransposition (Mulhardt et al., 1994; Takahara et al., 1996; van den Hurk et al., 2007; Vitullo et al., 2012). When L1 elements in zygotes are repressed immediately after fertilization, pre-implantation development is arrested at the two- and four-cells stages, leading to 50% reduction the rates of the blastocyst development and embryo fragmentation. It has shown that the L1 contributes to early embryo development and exits from the two-cells stage (Percharde et al., 2018; Jachowicz et al., 2017; Beraldi et al., 2006). Furthermore, researchers have found that overexpression of L1 causes abnormal chromatin decondensation, thus suggesting the another role of L1 transcript independent (Jachowicz et al., 2017).

Meanwhile, Alu elements, which belong to SINE family, are the most abundant repeats in the human genome, participating in regulating gene expression in early embryos. The studies have shown that 32 and 129 genes are unregulated in the transition from oocyte to four-cells stage and from four- to eight-cells stage, respectively (Tohonen et al., 2015) (Fig. 2c). Most of these genes have similar regulatory motifs that overlapped with Alu elements and these sequences appear in the 5’UTR, promoter, and around the transcription start site (TSS), which demonstrates that active Alu elements may regulate certain gene expression in human embryos.

Apart from the non-LTR retrotransposons, LTR elements also have indispensable roles in embryos. Although most LTRs have lost retroviral activity (Lower et al., 1996), stage-specific expression demarcates the different developmental stages and distinct cell populations in early embryos (Goke et al., 2015). Murine endogenous retrovirus-like gene (MuERV-L) which the most quickly transcribed gene from a zygotic genome, begins in the S phase of the first cell cycle and continues to blastula (Kigami et al., 2003). Compared with somatic tissues, the TSSs of two-cell-specific genes show preferential enrichment for MuERV-L and other ERV-L elements. Thus, MuERV-L is a potential marker of two-cell like cells because of the maximal transcription among LTRs. Transposition in cleavage-stage embryos is believed to use MuERV-L reverse transcriptase (Peaston et al., 2004; Macfarlan et al., 2012) (Fig. 2c). Disrupting the transcription of MuERV-L leads to a remarkable decrease in developmental competence after the four-cell stage, confirming its function in embryos (Kigami et al., 2003). In addition, existing researches have confirmed that LTRs not only promote some stage-dependent genes expression (such as *Rpl41* and *2610005H11Rik*) (Peaston et al., 2004) but also contribute to the accessibility of chromatin landscape in early embryos (Wu et al., 2016).

## Retrotransposons in PSCs

PSCs have the potential to self-renew and differentiate into three germ layer cell types (endodermal, mesodermal, and ectodermal cells) until adulthood (He et al., 2009; Hackett & Surani, 2014) (Fig. 2a). Transcription factors specifically expressed in PSCs (such as OCT4, SOX2, KLF4, C-MYC, and NANOG) control pluripotency, and they prefer to bind to functional genome features (such as promoters, enhancers) and open chromatin. Studies in recent years have shown that a large portion of human open chromatin regions (44%) overlap with TEs (Jacques et al., 2013) and transcription factors (such as OCT4, SOX2, and CTCF) are found bind to distinctive classes of TEs (Chen et al., 2008). On the other hand, some TEs work as promoters and enhancers (such as ERV) in PSCs. All the aforementioned evidence illustrates that retrotransposons provide regulation elements for core pluripotency transcription factors and thus may help maintain pluripotency.

It has been demonstrated that L1 full-length mRNA and the ORF1-encoded protein (L1TD1) can be detected in both ESCs and iPSCs, caused by an overall decrease in CpG methylation in the L1 promoter region (Wissing et al., 2012). L1TD1 is a stem-cell-specific RNA binding protein and chromatin immunoprecipitation sequencing (ChIP-seq) results show that pluripotency factors OCT4, SOX2, and NANOG all bind to the promoter of L1TD1, while deleting L1TD1 leads to the immediate down regulation of OCT4 and NANOG (Narva et al., 2012). Further research shows that more than half of de novo LINE-1 insertions are full length and enriched in the specific protein-coding genes of PSCs (Garcia-Perez et al., 2007; Klawitter et al., 2016) (Fig. 2c). Knockdown of L1 RNA inhibits mESC self-renewal (Percharde et al., 2018). Therefore, it is logical to assume that reactivation of LINE1 is required for pluripotency maintenance and self-renewal.

There are two states of pluripotency, naive and primed (Nichols & Smith, 2009). Each state has its own special morphology, gene expression pattern, and epigenetic modification, which can serve as methods of distinction. Some retrotransposons have been found to exhibit dramatic stem-cell-specific expression, and are considered as a marker of the naive state. LTR7-HERVH, a primate-specific endogenous retrovirus, was found to be enriched in the TSS of 127 HERVH-lincRNAs in both human ESCs and iPSCs, and 40% of them are annotated as enhancers in ESCs, whereas only 2.2% are annotated in other cells (Kelley & Rinn, 2012; Lu et al., 2014; Wang et al., 2014) (Fig. 2c). HERVH is considered a promoter because of the enrichment of H3K4me3, and it provides binding sites at 5’UTR for NANOG, with OCT4 and SOX2 (Goke et al., 2015; Wang et al., 2014; Loewer et al., 2010; Santoni et al., 2012). Recently, the study has reported that HERVH influences on chromatin architecture through establishing topologically associating domains (TAD) boundaries (Zhang et al., 2019). When knockout or transcriptional repression of HERVH in hESCs is achieved, corresponding TAD boundaries are eliminated and new chromatin domain boundaries rebuild when HERVH elements are inserted into the genome. In this process, transcription of genes upstream from the HERVH loci are affected, suggesting HERVH participates in hESC-specific gene regulation network (Zhang et al., 2019). Another naive pluripotency transcription factor LBP9 has been confirmed to work with HEVRH to drive hESC-specific pluripotency-modulating long non-coding RNAs, suggesting that HERVH elements also participate in pluripotency regulation and self-renewal development via lincRNA regulation (Kelley & Rinn, 2012; Wang et al., 2014).

Heterogeneity is another important phenotype in PSCs and is a key issue that remains unsolved. iPSCs, which are reprogramed from somatic cells, are highly similar to ESCs in transcription profiles but display a more open chromatin state (Cao et al., 2018). Despite the origin, almost all the repetitive elements have been found to be upregulated in mouse iPSCs compared to differentiated cells, such as L1. *ERV MusD* remains highly expressed, whereas other elements, such as *IAP*, *HERVK*, and *S71 (LTR6b)* display a fully repressed state, similar to expression patterns in ESCs (Fort et al., 2014; Friedli et al., 2014). Some of the elements are partially expressed in iPSCs, which are completely repressed in ESCs, suggesting a failure to reactivate sequence-specific repressors during reprogramming (Friedli et al., 2014) and shows heterogeneity is also associated with retrotransposon-selective transcription.

## Epigenetic regulation of retrotransposon

Although most retrotransposons have become inactive during evolution, some are still active and can insert new copies in the genome. Additionally, active retrotransposons are triggered in specific stages, especially in germlines, early embryos, and PSCs, which have a more open epigenetic environment. However, if somatic mutagenesis occurs as a result of diseases, cancer, or aging, the retrotransposons may be reactivated, and the abnormal activity may cause frequent transposition, breakage of DNA, and genome instability (Tubio et al., 2014). In response, cells have evolved mechanisms to keep retrotransposons elements tightly repressed (Vagin et al., 2006; Tam et al., 2008; Watanabe et al., 2008; Di Giacomo et al., 2013), and the regulation mechanisms can be divided into several levels: transcription, mRNA mature and export, translation, RNP formation, and transposition.

Epigenetic regulation occurs at transcriptional and translational levels. Silencing TEs are characterized by heterochromatin which is highly compact chromatin architecture, and are rich in repressive histone modifications, DNA methylation, and co-repressor proteins (such as NuRD complex, HP1, and repressive epigenetic enzymes). DNA methylation levels regulate retrotransposon expression in ESCs and iPSCs. DNA methylation enzymes (DNMT3a, DNMT3b, or DNMT1) are responsible for DNA methylation. Double knockout of DNMT3a/3b causes global loss of DNA methylation, and over 98% of the loss overlaps with repeat sequences (Li et al., 2015). The majority of ERVK subfamily members are sensitive to DNMT3a/3b, while DNMT1-mediated methylation activity is critical for suppressing IAP transcription in the mouse genome (Li et al., 2015). Additionally, the DNA methylation level of LINE1 promoter that contains some CpG sites, affects its transcription, and DNA hypomethylation of the promoter triggers its expression (Li et al., 2015). After fertilization, genome-wide DNA demethylation occurs in mammalian embryos. Thus, PSCs genomes are characterized by their low content of DNA methylation, which correlates with increasing levels of L1 mRNA expression (Gaysinskaya et al., 2018).

Repressive histone modifications are important in silencing retrotransposons. After knockout of H3K9me3 site methyltransferase Setdb1 in mESCs, ERV elements lose H3K9me3 accompanied by unregulated genes as 15% of promoters are proximal to demethylated ERVs (Karimi et al., 2011). Death domain associated protein (Daxx) binding sites are highly enriched in the IAP and ERVK. Daxx/Atrx complex can recruit Suv39h1 (another H3K9me3 site methyltransferase) to promote H3K9me3 and safeguards hypomethylated genome in naive pluripotent state (He et al., 2015). Similarly, histone chaperone CAF-1 mediates the replacement of H3.3 with H3.1/3.2 at retrotransposon regions, and this process is associated with deposition of repressive histone markers (H3K9me3, H3K9me2, H3K27me3, and H4K20me3). Subsequently, CAF-1 protects preimplantation mouse embryos from endogenous retrotransposons. Recently, a genome-wide CRIPSR-Cas9 screening was done in human cells, which aims to find genes that participant in L1 retrotransposition. Protein MORC2 and human silencing hub (HUSH) complex helped each other to repress the endogenous, evolutionarily young L1s in both hESCs and K562 cells, and they finally promoted deposition of H3K9me3 for L1s transcriptional silencing (Liu et al., 2018).

**Fig. 3:**
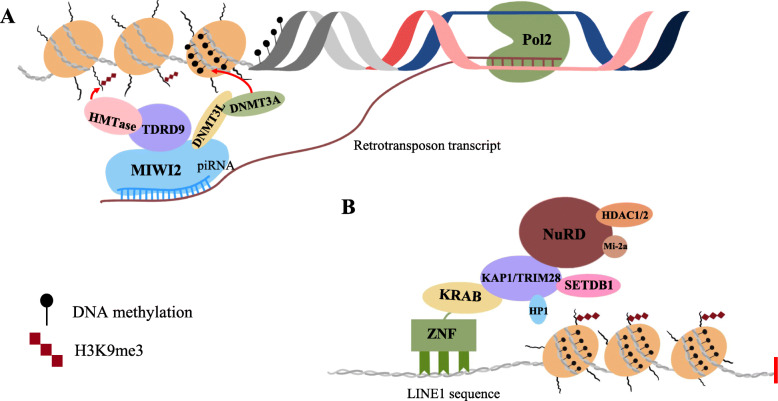
Epigenetic regulations of retrotransposons by DNA methylation and H3K9me3 modification. **a** Schematic diagram of different forms in retrotransposon repression. **a** piRNA-pathway is the most common mechanism to regulate retrotransposons in germ cells; piRNA guides PIWI protein to bind transposon transcripts, which have a matching sequence, and cleave target RNA. In addition, piRNAs work not only at the post-transcriptional level, but also regulate DNA methylation and histone modification. **b** KAP1/Trim28 complex is important in retrotransposons repression in early embryogenesis and ESCs. In this mechanism, KRAB-ZFPs recruit KAP1 to repress transcription of targeted retrotransposon, and KAP1 represses genes by recruiting histone methyltransferases SETDB1, HP1, and NuRD histone deacetylase complex

It is worth mentioning that histone and DNA methylation work in tandem and form a typical mechanism. In early embryogenesis and ESCs, KRAB domain-containing zinc finger proteins (KRAB-ZFPs) and KRAB-associated protein 1 (KAP1, also known as tripartite motif containing protein 28, TRIM28)-regulating retrotransposon silencing has been explored extensively. KRAB-ZFPs recognize specific retrotransposon sequences and recruit KAP1 to repress transcription of the targeted retrotransposon and adjacent genes. KAP1 repressed genes by recruiting histone methyltransferase SETDB1, HP1, and the NuRD histone deacetylase complex. KAP1 deletion decreased the level of H3K9me3, and upregulated ERVs, partial IAP elements, and genes such as *ZFP575*, *Prnp*, and *Serinc3*. Therefore, ESCs used KAP1-dependent pathway which resulting in H3K9me3 enrichment on retro-elements (Matsui et al., 2010; Rowe et al., 2010). Through this mechanism, we found that epigenetic silencing could spread from repetitive elements to neighboring genes (Fig. 3a).

In post-transcription regulation, the functions of silencing TEs by microRNA (miRNA), short interfering RNA (siRNA), and the P-element-induced wimpy testis (PIWI)-interacting RNA (piRNA) pathways have been confirmed (Fig. 3b). Tissue-specific siRNA and miRNA pathways are active in all tissues while the piRNA pathway is predominantly active in the germline (Slotkin & Martienssen, 2007; Ghildiyal & Zamore, 2009; Heras et al., 2013; Hamdorf et al., 2015). Genome-wide DNA demethylation occurs in mammalian embryos after fertilization. However, piRNA/Dnmt3L pathway is not responsible for suppressing TEs in this time (Rougier et al., 1998; Hajkova et al., 2002; Bourc'his & Bestor, 2004). In MII oocytes, piRNA is present in almost 50% of transcripts, reduces to about 25% (similar to miRNA), and finally drops to undetectable levels in ICM (Ohnishi et al., 2010). One-cell embryos injecting with mDicer-siRNA or long mDicer-dsRNA show a 50% increase in IAP and MuERV-L transcript abundance at 8-cells stage, suggesting the function of piRNA is progressively replaced by other small RNAs (Svoboda et al., 2004).With the development of pre-implantation mouse embryos, small RNA transcription changes from siRNA/piRNA to miRNA, but the post-transcriptional mechanism continues to be effective in repressing retrotransposons (Ohnishi et al., 2010; Russell et al., 2016).

## Conclusions and perspectives

TEs comprised a great part of the genome, but were previously considered useless. With more and more researches, we have recognized that few retrotransposons still work even though a majority of them have been truncated during genome evolution. Although retrotransposons are usually regulated by small RNAs, DNA methylation, and histone modifications, the global genome is in a more open state in PSCs compared to somatic cells, providing opportunities for repeat sequence transcription, and retrotransposons take part in gene regulation by actively reshaping chromatin structure or providing transcription factor binding sites.

The vast majority of retrotransposon functions are not very clear, but specific expression patterns of each family and subfamily and their effect on the genome can be observed. It is worth mentioning that different iPS cell lines have different transcription levels in some transposon classes, indicating heterogeneity of cell lines. Moreover, although ESCs and iPSCs share similar repetitive element expression, differences may exist due to variations in epigenetic modifications. As of yet, we still do not have a detailed protocol to evaluate a good iPS cell line. Retrotransposon transcription level may be considered as a criterion if it proves to work well in cell therapy. Furthermore, active or repressive types of transposon expression may be a new way to induce cells into the desired stage or condition, thus having potential in model construction and tissue differentiation.

Despite having benefits, the random insertion of transposons may cause genome instability and possibility of affecting promoter function, gene transcription, and pre-mRNA slicing, which happens more frequently in germ cells and early embryos because of the low level of repressive epigenetic regulation. LINE1 is associated with tumorigenesis in several cancers and human genetic diseases caused by Alu element homologous recombination (reach up to 0.3%) (Ahmed et al., 2010; Deininger & Batzer, 1999). By evaluating the expression of LINE1 elements, it was found that high levels of ORF1 occur more frequently in breast and ovarian cancer (Chen et al., 2012; Rodic et al., 2014), whereas ORF2 expression often occurs earlier in the tumorigenesis process (De Luca et al., 2016), indicating that retrotransposons may be a useful early diagnostic marker of certain cancers. However, translation of transposons can also be used as a tool. Transformation efficiency is improved by using hyperactive PiggyBac transposase in insect (Eckermann et al., 2018), and CRISPR/Cas9 gene editing system also can be stably transformed in multiple human cells, including iPSCs (Ishida et al., 2018; Schertzer et al., 2019), providing a new platform for genetic disease treatment.

Aberrant transposon activation leads to genome instability and carcinogenesis, thus, the mechanism of transposons regulation needs to be analyzed in detail. Apart from the classic regulation mechanism, more and more epigenetic modifications have been discovered, such as histone succinylation, crotonylation, 6 mA DNA and m6A mRNA modifications. As there are no detailed studies about the relationship between these new modifications and TE regulation, more regulation mechanisms need to be elucidated.

To date, many TEs are still not recognized, and the sequences may have changed with genome evolution. Functions of enhancers are not easy to detect because of the complex spatial structures of chromatin. TEs are a large family and have multiple functions and with the development of new technologies, we will be one step closer to understanding them and their regulation.

## Abbreviations

ESCs: Embryonic stem cells
iPSCs: Induced pluripotent stem cells
hPSCs: Human pluripotent stem cells
LTRs: Long terminal repeats
ERVs: Endogenous retroviruses
IAP: Intracisternal A particle
ORF: Open reading frames
L1: LINE1–1
SINEs: Short interspersed nuclear elements
LINEs: Long interspersed nuclear elements
L1Md: *Mus musculus* domesticus L1 retrotransposon
ICM: Inner cell mass
TE: Transposable element
ChIP-seq: Chromatin immunoprecipitation sequencing
piRNAs: P-element-induced wimpy testis (PIWI)-interacting RNAs
TSS: Transcription start site
MuERV-L: Murine endogenous retrovirus-like gene
NHEJ: Non-homologous end joining
Daxx: Death Domain Associated Protein
KRAB-ZFPs: KRAB domain-containing zinc finger proteins
KAP1: KRAB-associated protein 1
siRNA: short interfering RNA
miRNA: microRNA
